# Assessing nutrition and other claims on food labels: a repeated cross-sectional analysis of the Canadian food supply

**DOI:** 10.1186/s40795-017-0192-9

**Published:** 2017-09-08

**Authors:** Beatriz Franco-Arellano, Jodi T. Bernstein, Sheida Norsen, Alyssa Schermel, Mary R. L’Abbé

**Affiliations:** Department of Nutritional Sciences, Faculty of Medicine, University of Toronto, FitzGerald Building, 150 College Street, Rm 315, Toronto, ON M5S 3E2 Canada

**Keywords:** Nutrition claims, Food labelling, Canada

## Abstract

**Background:**

In 2010, nutrition claims were investigated in Canadian foods; however, many nutrition and other claims have been introduced since then. This study aimed to determine: i) the proportion of foods carrying claims in 2013, ii) the types and prevalence of nutrition claims (nutrient content claims, health claims, general health claims) and other claims displayed on labels in 2013, iii) and trends in use of nutrition claims between 2010 and 2013.

**Methods:**

Repeated cross-sectional analysis of the University of Toronto Food Label Information Program (FLIP) of Canadian foods (2010/11 *n* = 10,487; 2013 *n* = 15,342). Regulated nutrition claims (nutrient content, health claims) were classified according to Canadian regulations. A decision tree was used to classify non-regulated general health claims (e.g., front-of-pack claims). Other claims (e.g., gluten-free) were also collected. Proportions of claims in 2013 were determined and χ^2^ was used to test significant differences for different types of claims between 2010 and 2013.

**Results:**

Overall, 49% of products in 2013 displayed any type of claim and 46% of foods in FLIP 2013 carried a nutrition claim (nutrient content claim, health claim, general health claim). Meal replacements and fruits/fruits juices were the categories with the largest proportion of foods with claims. At least one approved nutrient content claim was carried on 42.9% of products compared to 45.5% in 2010 (*p* < 0.001). Health claims, specifically disease risk reduction claims, were slightly lower in 2013 (1.5%) compared to 1.7% in 2010 (*p* = 0.225). General health claims, specifically front-of-pack claims, were carried on 20% of foods compared to 18.9% in 2010 (*p* = 0.020). Other claims, specifically gluten-free, were present on 7.3% of foods.

**Conclusions:**

Nutrition and other claims were used on half of Canadian prepackaged foods in 2013. Many claims guidelines and regulations have been released since 2010; however, little impact has been seen in the prevalence of such claims in the food supply. Claims related to nutrients of public health priority, such as sugars and sodium, were not commonly used on food labels. Monitoring trends in the use of nutrition and other claims is essential to determine if their use on food labels reflects public health objectives, or instead are being used as marketing tools.

## Background

**Fig. 1:**
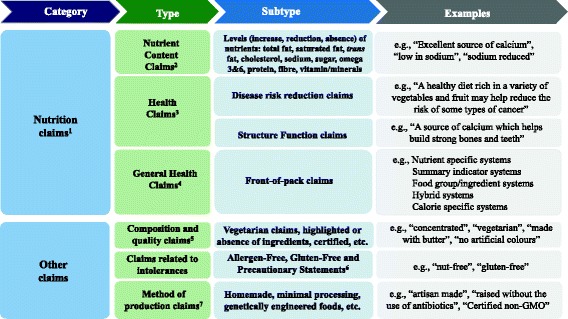
Overview of the types of nutrition and other claims displayed on food labels in the Canadian food supply^1^. 1. Classified according to Canadian regulations (sections B.01.503 to B.01.513 and B01.601 to B01.603 of the Food and Drug Regulations), Health Canada and Canadian Food Inspection Agency [13, 15, 66–69]. 2. Classified according to Canadian Regulations (sections B.01.503 to B.01.513 of the Food and Drug Regulations) [13]. 3. Classified according to Canadian regulations (sections B01.601 to B01.603 of the Food and Drug Regulations) [13]. 4. Claims not specifically regulated by Government [69]. 5. Composition and quality claims classified according to the Canadian Food Inspection Agency [67]. 6. Allergen-free and gluten-free claims classified according to the Canadian Food Inspection Agency [68]. 7. Methods of production claims classified according to the Canadian Food Inspection Agency [70]

Poor diets, characterized by the inclusion of processed foods with excessive salt, fat and sugar, are major risk factors for chronic non-communicable diseases (NCDs) such as obesity, type 2 diabetes, cardiovascular diseases (CVDs) and some cancers [1, 2]. Nutrition labelling has been included by the World Health Organization as an intervention strategy to provide consumers meaningful information on the nutritional content of foods and to help them select more healthier ones [2, 3]. One can argue that well-designed food labels, with accurate and easy-to-understand nutrition information, can have the potential to nudge consumers towards informed healthy food choices [4–8]; although, others have suggested that nutrition claims are being used more as marketing tools by industry [8, 9]. Moreover, products with nutrition claims on food labels could be perceived as “healthier” by consumers [10]. In many countries, nutrition claims are subject to regulations. Organizations such as the CODEX Alimentarius [11] and the European Union [12] have provided a common basis towards the standardized use of nutrition claims across countries. In Canada, mandatory nutrition labelling on pre-packaged food products was introduced in 2003 [13] and updated in late 2016 [14], requiring manufacturers to provide nutrition information (Nutrition Fact table and ingredients list). It also established requirements for the voluntary use of nutrition claims (Fig. 1) including: *nutrient content claims*, which describe the amount of a nutrient in a food [15], such as “low in sodium” or “very high in fibre”, and *health claims*, which are statements about the healthful effects of a certain food or food constituents consumed within a healthy diet on a person’s health, and which comprise the following subtypes: disease risk reduction claims (e.g., “a healthy diet rich in a variety of vegetables and fruit may help reduce the risk of some types of cancer”), and structure function claims (e.g. “vitamin A aids in the development and maintenance of night vision”) [16]. Other *general health claims*, which broadly include front-of-pack claims, “healthy” claims, symbols, logos, or check marks found on labels are not specifically regulated by the Government in Canada, although such claims must be truthful and not mislead consumers [15]. Nutrition labelling and nutrition claims regulations can change when new scientific findings and developments worldwide emerge. In Canada, for example, new rules and guidelines have been published in past years including specific guidance by Health Canada on health claims [17], the use of probiotics in foods [18], sodium reduction targets [19], position on gluten-free claims [20], along with a number of non-Government led front-of-pack claims such as the Whole Grains Stamp [21], calorie specific systems, and even the discontinuation of others, such as the Heart and Stroke Foundation’s Health Check™ symbol [22]. In addition to nutrition claims, other claims (Fig. 1) including those associated with intolerances such as gluten-free, dietary practices (e.g., vegetarian) or “natural” are being used as wholesome nutrition indicators [23]. Thus, there is a need to investigate if the use of nutrition and other claims on food labels has been affected by the advent of these new government policies and guidelines, as well as industry-led initiatives. The objectives of this study were to determine: i) the proportion of foods carrying claims in FLIP 2013, ii) the types and prevalence of nutrition claims (nutrient content claims, health claims, general health claims) and other claims displayed on labels in 2013, iii) and trends in use of nutrition claims between 2010 and 2013.

## Methods

### Food label information program overview, data collection and data processing

The Food Label Information Program (FLIP) database was developed at the University of Toronto and contains information found on the labels of Canadian pre-packaged food and beverage products (e.g. nutrition information, ingredient lists, presence of nutrition marketing, price, brand, container size, UPC code) [24]. FLIP is updated approximately every three years, which enables periodic evaluation of the nutrient content and nutritional quality of the Canadian food supply. Two FLIP collections have been completed, one in 2010/11 (*n* = 10,487) and the second in 2013 (*n* = 15,342), as described elsewhere [24, 25]. A 2017 collection is currently underway. Label information was collected from the major Canadian grocery retailers (Loblaws, Metro, Sobeys and Safeway in 2013), which together represents approximately 75% of the grocery retail market share [26]. Information was collected for one size of all products with a Nutrition Facts table (including all flavour variations), and included national and private label brands. Products were excluded from collection if they were seasonal products, Natural Health Products (such as herbal remedies or homeopathic medicines), baby and toddler foods, and products without a Canadian Nutrition Fact table. FLIP 2010 and 2013 were collected using a similar approach; however, in 2013 the food collection and data storage were fully digitalized, whereas in 2010 much of the collection was manual [25]. Data collection software (programmed for the iPhone) was developed to scan barcodes and take photos of all sides of the package in-store. The bar codes of each product were scanned first to determine if the food had already been collected at another store to prevent duplicate collection [25]. Product information was then uploaded to a website where additional information was entered about each product [25]. Later, trained staff classified foods using several classification including Schedule M of the Food and Drug Regulations with 22 pre-defined food categories and 153 subcategories, in force at the time of the collection [13, 27]. Schedule M reference serving amounts are a specific regulated quantity of a type of food usually eaten by an individual at one sitting and which serve as the basis of compositional criteria for nutrient content claims and health claims [27]. An additional category for meal replacements was also included. All products were also categorized according to Health Canada’s sodium categories [19, 25]. Lastly, the nutrition information for products requiring preparation (e.g., muffin mix, condensed soups) was calculated according to package instructions, using ESHA Food Processor software and food composition data from the Canadian Nutrient File (CNF) [28] to allow for standardized comparisons within a food category [25].

### Nutrition claims classification and validation

Nutrition claims on food labels collected in FLIP 2013 were classified according to the types and subtypes defined in the previous data collection [24]. See Fig. 1 for an overview of the different types of claims. All Government-approved variations in wording were included for each claim:

- **Nutrient content claims** (NCC) which are claims, as mentioned earlier, that state the amount (including presence, reduction or absence) of total fat, *trans* fat, saturated fat, cholesterol, vitamin and minerals, sugars, sodium, polyunsaturated fatty acids (omega-3 and omega-6), fibre, protein, energy and lean claims [13, 15];
- **Health claims**, which are statements about the healthful effects of a certain food or food constituents consumed within a healthy diet on a person’s health, including [13, 16]:

- ▪ **disease risk reduction claims** (DRRC) with respect to sodium and hypertension; calcium and osteoporosis; dietary fat, saturated fat, cholesterol, *trans* fatty acids and coronary heart disease; fruits, vegetables, and cancer for both collections. Additionally, information on the following new claims were documented in FLIP 2013 as they were approved after 2010: plant sterols and cholesterol lowering; oat products and cholesterol lowering; psyllium products and cholesterol lowering; unsaturated fat and cholesterol lowering; barley products and cholesterol lowering.
- ▪ **structure/function claims** (e.g. “vitamin A aids in the development and maintenance of night vision”).

c) **‘General health claims’** [15], specifically front-of-pack (FOP) claims by specific subtype: nutrient specific systems (NSS), summary indicator systems (SIS), food group/ingredient systems (FGIS), and hybrid systems (HS) were classified as described earlier [24, 29]. In addition, calorie specific systems (CSS) were included since they have appeared on labels after 2010.

**Fig. 2:**
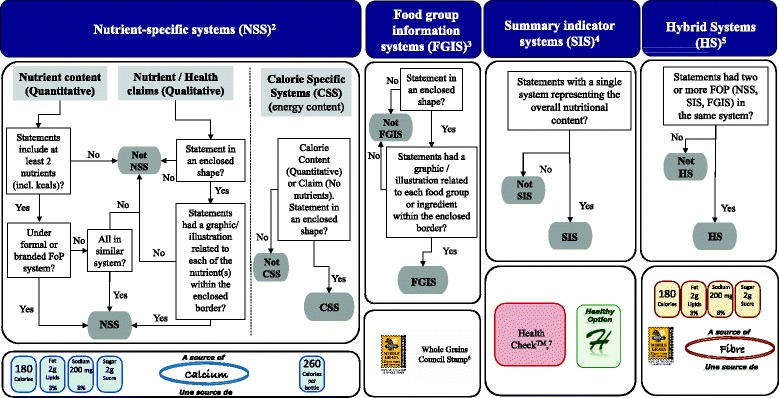
Decision tree framework used to classify Front-of-Pack^1^ (FOP) claims in FLIP 2013 and some examples. 1. Front-of-Pack claims are not specifically regulated in the Canadian Food and Drug regulations; therefore, the following definition was adopted: “Systems that use nutrient criteria and symbols to indicate that a product has certain nutritional characteristics. Symbols are often placed on the principal display panel of the product, but may also be found on the side, top, or back panels or on self tags”. Adapted from Schemel et al. and The National Academies of Sciences, Engineering and Medicine (formerly Institute of Medicine) [24, 31]. 2. Nutrient-specific systems (NSS): Use symbols to display the amount of select nutrients or calories per serving. 3. Food group information systems (FGIS): Use symbols to convey the presence of a food group or ingredient. 4. Summary indicator systems (SIS): Use symbols to provide summary information on the nutrient content of a food using a single symbol/score based on nutrient thresholds or algorithm. 5. Hybrid Systems (HS): Use symbols identify where two or more of the NSS, FGIS or SIS were displayed. 6. Whole Grains Council’s Whole Grain Stamp™. Reprinted with permission https://wholegrainscouncil.org. 7. Heart and Stroke Foundation’s Health Check™. Reprinted with permission http://www.heartandstroke.ca

Claims classifications previously entered by staff were validated by members of the research team (JTB, SN, BFA, or AS) in late 2015 and 2016. Claims that had defined regulations and approved wording (nutrient content claims, disease risk reduction claims) were classified as indicated in the Food and Drug Regulations (FDR) [13]. To validate front-of-pack classifications (a subtype of general health claims, see Fig. 1) and where specific regulations are not defined in Canada, a decision tree for front-of-pack was developed for FLIP 2013 by a research team member (JTB) and adjusted by another research member (BFA) to reduce subjective bias regarding the categorization of these types of claims (Fig. 2). The decision tree was based on methodology previously published [24, 29–31]. Briefly, these classifications included the use of symbols to convey information on the amount of select nutrients (e.g., vitamins or minerals) or calories per serving, the presence of a food group or ingredient (e.g., whole grain, milk), summary information on the overall nutrition profile of a food using a single symbol/score, and hybrid systems when two or more of the previous front-of-pack systems were displayed [24, 29–31].

Ten percent of the nutrition marketing on food labels was analyzed a second time by two research members (JTB, BFA) for inter-person reliability. Discrepancies in the nutrition claims classifications were discussed by team members and a final decision was agreed upon. Lastly, a brand review was performed by one research member (BFA) to ensure consistency by brand when more than one discrepancy was found at a brand-specific level, to minimize classification errors and, to ensure all products within a brand were classified consistently.

### Other claims classification and validation

Claims related to intolerances and dietary practices (e.g., gluten-free), were captured for the first time in FLIP 2013. Gluten-free claims, for example, have appeared recently on labels due to a growing demand from consumers and a response from manufacturers to meet their demands [32]. Thus, these claims were deemed of high importance to investigate. Gluten-free claims were classified and validated as “gluten-free” if a declaration was made on package.

### Statistical analyses

Products from FLIP 2013 (*n* = 15,342) were excluded for analysis if they were natural health products [33], such as herbal remedies or homeopathic medicines (*n* = 1), or had an incorrect nutrient declaration as determined by Atwater calculations (*n* = 55). The Atwater factor is a theoretical energy conversion factor for foods [34]. Products with a difference of 20% or more on the declared values of calories vs. calculated were excluded to be consistent with the Canadian Food Inspection Agency’s nutrition labelling compliance test tolerance, which requires a limit of 20% for nutrient accuracy for energy on the Nutrition Facts table [35]. The final number of products analyzed in this study was *n* = 15,286. Descriptive statistics (frequencies) were calculated using IBM SPSS version 24 (IBM Corporation), and reported as:

i) Proportion of products carrying claims in FLIP 2013, overall and per Schedule M food categories [13, 27].
ii) Types and prevalence of nutrition and other claims in FLIP 2013:
  - Nutrition claims
    ▪ Proportion of products carrying nutrient content claims, overall and per type of nutrient content claim, including presence, reduction or absence.
    ▪ Proportion of products carrying health claims, specifically disease risk reduction claims, overall and per type of disease risk reduction claim. Structure-function claims were collected, but not analyzed in this study.
    ▪ Proportion of products carrying general health claims, specifically front-of-pack claims, overall and per type of front-of-pack claim.
  - Overall prevalence of other claims, specifically gluten-free claims
iii) Trends in use of nutrition claims between 2010 and 2013. Results from FLIP 2013 were compared to FLIP 2010 [24]. Pearson χ^2^ was used to determine significant differences in the proportion (number of claims weighted to the number of products collected in each data set) within each major claim category (nutrient content claims, disease risk reduction claims, front-of-pack claims) and in each individual type of claim (e.g., total fat, sodium, front-of-pack nutrient specific systems, etc).

## Results

i) **Proportion of foods carrying claims in FLIP 2013**

Findings from this study showed that overall 49% of products displayed some type of claim on food labels. During label review, 76 products (0.5%) had discrepancies in their nutrition claim classifications that required further review by research team members. Food categories with the largest proportion of foods in FLIP 2013 were bakery (13.6%), combination dishes (8.9%), dairy products and substitutes (8.1%), sauces/dips/gravies/condiments (8.0%), and fruit and fruit juices (7.1%), as shown in Table 1. However, the food categories with the largest proportion of foods carrying claims on food labels were meal replacements (96.4%), fruits and fruits juices (68.5%), dairy products (64.4%), snacks (62.1%) and soups (61%) (Table 1). For comparison purposes, number of products collected per Schedule M food categories in FLIP 2010 is presented in Table 1 [24].

**Table 1:** Proportion of products in FLIP 2010-2013 and proportion of foods carrying any claim in FLIP 2013

Schedule M Category^a^	FLIP 2010^b,c^		FLIP 2013		Products with any claim in FLIP 2013	
	*n*	%	*n*	%	*n*	%
Bakery Products	1636	15.6%	2084	13.6%	1072	51.4%
Combination Dishes	1044	10.0%	1357	8.9%	530	39.1%
Dairy Products and Substitutes	839	8.0%	1240	8.1%	799	64.4%
Sauces, Dips, Gravies and Condiments	691	6.6%	1229	8.0%	333	27.1%
Fruits and Fruit Juices	800	7.6%	1089	7.1%	746	68.5%
Cereals and Other Grain Products	777	7.4%	988	6.5%	592	59.9%
Meat, Poultry, Their Products and Substitutes	643	6.1%	895	5.9%	426	47.6%
Vegetables	623	5.9%	834	5.5%	338	40.5%
Desserts	525	5.0%	827	5.4%	403	48.7%
Snacks	471	4.5%	794	5.2%	493	62.1%
Sugars and Sweets	235	2.2%	749	4.9%	161	21.5%
Fats and Oils	476	4.5%	535	3.5%	289	54.0%
Beverages	257	2.5%	482	3.2%	231	47.9%
Soups	334	3.2%	456	3.0%	278	61.0%
Miscellaneous category	198	1.9%	450	2.9%	163	36.2%
Marine and Fresh Water Animals	336	3.2%	440	2.9%	211	48.0%
Nuts and Seeds	109	1.0%	220	1.4%	116	52.7%
Legumes	189	1.8%	180	1.2%	99	55.0%
Potatoes, Sweet Potatoes and Yams	95	0.9%	140	0.9%	76	54.3%
Dessert Toppings and Fillings	55	0.5%	116	0.8%	37	31.9%
Salads	60	0.6%	70	0.5%	28	40.0%
Egg and Egg Substitutes	37	0.4%	56	0.4%	31	55.4%
Meal Replacements	57	0.5%	55	0.4%	53	96.4%
TOTAL	10,487	100.0%	15,286	100.0%	7505	49.1%

^a^Food categories defined as per Schedule M of the Food and Drug Regulations [13, 27]

^b^Number and proportion of foods in FLIP 2010 (*n* = 10,487), as published in *Schermel* et al.*, Appl Physiol Nutr Metab 2013;38(6):666-672* [24]

^c^Products with any claim per food category were not reported for FLIP 2010 and therefore were not included in this paper

ii) **Types and prevalence of nutrition and other claims in FLIP 2013**

- **Nutrition claims**

Forty-six percent of foods carried at least one nutrition claim. This value did not include products that carried only gluten-free claims, as gluten is not considered a “nutrient”.

- ▪ *Nutrient content claims*

At least one Health Canada approved nutrient content claim was carried on 42.9% of products. The percentage of products sold with each type of nutrient content claim, as well as the top five food categories with the highest proportion of these claims in FLIP 2013, are shown in Table 2. For comparison purposes, data for the different Schedule M food categories that displayed nutrient content claims in 2010 [24] are also presented in Table 2.

**Table 2:** Proportion of products carrying nutrient content claims per Schedule M^a^ food categories in FLIP 2010-2013

Nutrient Content Claims^b,c^	FLIP 2010^d^				FLIP 2013^f^				*p* value overall^g^
	Top food categories carrying nutrient content claims in FLIP 2010^a,e^				Top food categories carrying nutrient content claims in FLIP 2010^a,e^				
	*n*	%	Food Category^a^	%	*n*	%	Food Category^a^	%	
Vitamins and minerals	1512	14.4%	Meal Replacements	42.1%	2319	15.2%	Meal Replacements	87.3%	0.095
*Any vitamin or mineral claim containing the following: “contains”, “source of”, “contains X essential nutrients”, “high in”, “higher in...”, “reduced”, “free”*			Fruit and Fruit Juices	34.0%			Fruit and Fruit Juices	48.8%	
			Dairy Products and Substitutes	30.0%			Dairy Products and Substitutes	43.8%	
			Cereals and Other Grain Products	27.9%			Cereals and Other Grain Products	25.2%	
			Vegetables	23.9%			Vegetables	19.4%	
Total fat	1631	15.6%	Soups	53.0%	1828	12.0%	Soups	40.1%	<0.001
*Free of fat, low in fat, reduced in fat, lower in fat, (%) fat free, no added fat*			Cereals and Other Grain Products	29.3%			Desserts	26.2%	
			Egg and Egg Substitutes	27.0%			Egg and Egg Substitutes	23.2%	
			Desserts	26.9%			Dairy Products and Substitutes	21.9%	
			Dairy Products and Substitutes	25.3%			Cereals and Other Grain Products	20.9%	
Trans fat	1622	15.5%	Potatoes, Sweet Potatoes and Yams	46.3%	1710	11.2%	Potatoes, Sweet Potatoes and Yams	37.1%	<0.001
*Free of trans fat, reduced in trans fat*			Snacks	44.2%			Snacks	33.1%	
			Bakery Products	36.4%			Bakery Products	26.5%	
			Fats and Oils	28.2%			Fats and Oils	24.7%	
			Miscellaneous category	26.3%			Salads	21.4%	
Fibre	1039	9.9%	Legumes	47.6%	1249	8.2%	Legumes	43.3%	<0.001
*Source of fibre, high source of fibre, very high source of fibre, more fibre*			Cereals and Other Grain Products	38.7%			Cereals and Other Grain Products	33.5%	
			Soups	18.6%			Combination Dishes	13.3%	
			Snacks	15.9%			Snacks	13.0%	
			Bakery Products	13.4%			Nuts and Seeds	12.3%	
Saturated fat	908	8.7%	Potatoes, Sweet Potatoes and Yams	33.7%	893	5.8%	Potatoes, Sweet Potatoes and Yams	27.1%	<0.001
*Free of saturated fat, low in saturated fat, reduced in saturated fat, lower in saturated fat*			Fats and Oils	27.6%			Fats and Oils	24.5%	
			Meal Replacements	24.6%			Salads	17.1%	
			Bakery Products	18.2%			Bakery Products	13.8%	
			Snacks	14.7%			Marine and Fresh Water Animals	12.7%	
Cholesterol	676	6.5%	Egg and Egg Substitutes	27.0%	803	5.3%	Egg and Egg Substitutes	23.2%	<0.001
*Free of cholesterol, low in cholesterol, reduced in cholesterol, lower in cholesterol*			Potatoes, Sweet Potatoes and Yams	25.3%			Potatoes, Sweet Potatoes and Yams	21.4%	
			Fats and Oils	18.3%			Fats and Oils	16.4%	
			Bakery Products	15.6%			Bakery Products	11.7%	
			Snacks	9.1%			Snacks	7.8%	
Sugar	418	4.0%	Fruit and Fruit Juices	27.3%	785	5.1%	Fruit and Fruit Juices	30.8%	<0.001
*Free of sugars, reduced in sugars, lower in sugars, no added sugars*			Beverages	13.6%			Beverages	17.0%	
			Sugars and Sweets	5.1%			Nuts and Seeds	9.5%	
			Desserts	4.8%			Dairy Products and Substitutes	7.1%	
			Fats and Oils	4.8%			Desserts	6.4%	
Sodium	474	4.5%	Soups	17.7%	755	4.9%	Nuts and Seeds	24.1%	0.121
*Free of sodium, low in sodium, reduced in sodium, lower in sodium, no added sodium, lightly salted*			Cereals and Other Grain Products	14.9%			Soups	19.5%	
			Nuts and Seeds	11.0%			Cereals and Other Grain Products	11.5%	
			Potatoes, Sweet Potatoes and Yams	7.4%			Vegetables	9.4%	
			Vegetables	7.1%			Snacks	8.4%	
Protein	221	2.1%	Egg and Egg Substitutes	13.5%	450	2.9%	Meal Replacements	43.6%	<0.001
*Source of protein, excellent source of protein, more protein*			Marine and Fresh Water Animals	9.5%			Egg and Egg Substitutes	17.9%	
			Meat, Poultry, Their Products and Substitutes	9.2%			Dairy Products and Substitutes	13.6%	
			Dairy Products and Substitutes	9.2%			Salads	12.9%	
			Meal Replacements	7.0%			Meat, Poultry, Their Products and Substitutes	11.7%	
Polyunsaturated fatty acids (PUFAs)	346	3.3%	Marine and Fresh Water Animals	30.4%	445	2.9%	Marine and Fresh Water Animals	31.8%	0.076
*Source of omega-3 PUFAs, source of omega-6 PUFAs*			Egg and Egg Substitutes	21.6%			Fats and Oils	17.8%	
			Fats and Oils	19.8%			Egg and Egg Substitutes	16.1%	
			Salads	5.0%			Salads	11.4%	
			Bakery Products	3.1%			Nuts and Seeds	7.7%	
Energy/Calories	261	2.5%	Beverages	27.6%	387	2.5%	Meal Replacements	29.1%	0.829
*Free of energy, low in energy, reduced in energy, lower in energy, source of energy, more energy, light in energy*			Meal Replacements	19.3%			Beverages	26.6%	
			Fats and Oils	6.5%			Fats and Oils	6.5%	
			Sugars and Sweets	6.4%			Sugars and Sweets	3.7%	
			Desserts	4.0%			Fruit and Fruit Juices	3.2%	
Lean	86	0.80%	Meat, Poultry, Their Products and Substitutes	12.9%	69	0.5%	Meat, Poultry, Their Products and Substitutes	7.4%	<0.001
Lean, extra lean			Snacks	0.2%			Marine and Fresh Water Animals	0.7%	

^a^Food categories defined as per Schedule M of the Food and Drug Regulations [13, 27]

^b^All approved variations in wording were included

^c^Classified according to Canadian regulations (sections B.01.503 to B.01.513 of the Food and Drug Regulations) [13]

^d^Number and proportion of foods carrying each type of nutrient content claim in FLIP 2010 (*n* = 10,487), as reported in *Schermel* et al.*, APNM, 2013;38(6):666-672* [24]

^e^Proportion of foods carrying a nutrient content claim in the top Schedule M food categories

^f^Number and proportion of foods carrying each type of nutrient content claim in FLIP 2013 (*n* = 15,286)

^g^Statistical difference in the overall proportion of foods carrying nutrient content claims between 2010 and 2013. A *p* value of <0.05 was considered statistically significant

- ▪ *Health claims*

This research only analyzed one subtype of health claims: disease risk reduction claims, which represented 1.5% of foods in FLIP 2013. Of the total number of products carrying these claims (*n* = 226), 89.3% of food labels (*n* = 202) carried one disease risk reduction claim, while the remaining products (*n* = 24) carried two disease risk reduction claims, mostly on cereals products. The disease risk reduction claims most commonly used together were related to: i) dietary fat, saturated fat, cholesterol, *trans* fatty acids and coronary heart disease, and ii) oat products and cholesterol lowering. Table 3 presents the prevalence of use for each disease risk reduction claim that has been approved by Health Canada up to 2013, as well as the top food categories that carried the largest proportion of disease risk reduction claims. For comparison purposes, the different Schedule M food categories that displayed disease risk reduction claims in 2010 [24] are also presented in Table 3.

**Table 3:** Proportion of products carrying disease risk reduction claims per Schedule M^a^ food categories in FLIP 2010-2013

Disease Risk Reduction Claims^b,c^	FLIP 2010^d,e^		FLIP 2013^g^				*p* value overall^i^
	Top food product categories with disease risk reduction claims in FLIP 2010^a,f^		Top food product categories with disease risk reduction claims in FLIP 2013^a,h^				
	%	Food category^a^	*n*	%	Food category^a^	%	
Sodium and hypertension	0.10%	Cereals and Other Grain Products	11	0.07%	Cereals and Other Grain Products	0.71%	0.199
		Fruit and Fruit Juices			Vegetables	0.36%	
Calcium and osteoporosis	0.10%	Fruit and Fruit Juices	8	0.05%	Fruit and Fruit Juices	0.37%	0.128
		Meal replacements			Dairy Products and Substitutes	0.32%	
Dietary fat, saturated fat, cholesterol, trans fatty acids and coronary heart disease	1.10%	Cereals and Other Grain Products	106	0.69%	Cereals and Other Grain Products	4.76%	<0.001
		Bakery Products			Fats and Oils	2.99%	
		Fats and Oils			Soups	2.85%	
Fruits, vegetables and cancer	0.50%	Fruit and Fruit Juices	65	0.43%	Fruit and Fruit Juices	3.49%	0.283
		Vegetables			Vegetables	3.00%	
					Legumes	1.11%	
Plant sterols and cholesterol lowering	-		9	0.06%	Fats and Oils	0.75%	-
					Fruit and Fruit Juices	0.46%	
Oat products and cholesterol lowering	-		49	0.32%	Cereals and Other Grain Products	4.45%	-
					Bakery Products	0.24%	
Pysllium products and cholesterol lowering	-		1	0.01%	Cereals and Other Grain Products	0.10%	-
Unsaturated fat and cholesterol lowering	-		0	0.00%	n/a		-
Barley products and cholesterol lowering	-		1	0.01%	Cereals and Other Grain Products	0.10%	-

^a^Food categories defined as per Schedule M of the Food and Drug Regulations [13, 27]

^b^All approved variations in wording were included

^c^Classified according to Canadian regulations (sections B01.601 to B01.603 of the Food and Drug Regulations [13])

^d^Proportion of foods carrying each type of disease risk reduction claim in FLIP 2010 (*n* = 10,487), as reported in Schermel et al., Appl Physiol Nutr Metab 2013;38(6):666-672 [24]

^e^Number of foods carrying each disease risk reduction claim was not reported for FLIP 2010 and therefore not included in this paper

^f^Proportion of foods carrying a disease risk reduction claim within each Schedule M food category was not reported for FLIP 2010 and therefore not included in this paper

^g^Number and proportion of foods carrying each type of disease risk reduction claim in FLIP 2013 (*n* = 15,286)

^h^Proportion of foods carrying a disease risk reduction claim in the top Schedule M food categories in FLIP 2013

^i^Statistical difference in the overall proportion of foods carrying disease risk reduction claims between 2010 and 2013. A *p* value of <0.05 was considered statistically significant

- ▪ *General health claims*

In this study, the only type of general health claims analyzed was front-of-pack claims (Fig. 1). Overall, 20% of foods carried at least one front-of-pack claim. Table 4 shows the frequency of use of front-of-pack claims and the top food categories that carried the most front-of-pack claims. As presented in the previous tables, the different Schedule M food categories that displayed front-of-pack claims in 2010 [24] are presented in Table 4.

**Table 4:** Proportion of products carrying front-of-pack claims per Schedule M^a^ food categories in FLIP 2010-2013

Front-of-Pack claims	FLIP2010^b,c^			FLIP 2013^d^				*p* value overall^f^
	Top food categories carrying front-of-pack claims in FLIP 2010^a,e^			Top food categories carrying front-of-pack claims in FLIP 2013^a,e^				
	%	Schedule M food categories^a^	%	*n*	%	Schedule M food categories^a^	%	
Nutrient Specific Systems	4.9%	Meal Replacements	14.0%	1036	6.8%	Cereals and Other Grain Products	18.1%	<0.001
*Systems with symbols that display the amount per serving of select nutrients or use symbols based on nutrient content claim criteria.*		Cereals and Other Grain Products	12.9%			Potatoes, Sweet Potatoes, and Yams	12.9%	
		Potatoes, Sweet Potatoes, and Yams	8.4%			Soups	10.5%	
		Nuts and Seeds	8.3%			Fruit and Fruit juices	8.8%	
		Desserts	7.6%			Bakery Products	8.5%	
Summary Indicator Systems	7.5%	Egg and Egg Substitutes	46.0%	726	4.7%	Egg and Egg Substitutes	39.3%	<0.001
*Systems with a single symbol, icon, or score that provide summary information about the nutrient content of a product.*		Soups	18.9%			Fruit and Fruit juices	13.9%	
		Fruit and Fruit juices	14.0%			Soups	11.0%	
		Bakery Products	10.3%			Combination Dishes	7.1%	
		Combination Dishes	9.5%			Vegetables	7.0%	
Food Group/Ingredient Systems	3.5%	Cereals and Other Grain Products	12.2%	726	4.7%	Cereals and Other Grain Products	17.2%	<0.001
*Systems with symbols based on the presence of a food group or food ingredient.*		Fruit and Fruit juices	6.4%			Fruit and Fruit juices	11.5%	
		Vegetables	5.8%			Bakery Products	8.8%	
		Snacks	5.5%			Snacks	7.7%	
		Bakery Products	4.2%			Vegetables	5.0%	
Hybrid Systems	7.0%	Legumes	11.6%	728	4.8%	Legumes	16.1%	<0.001
*Systems where two or more of the NSS, FGIS or SIS were displayed.*		Egg and Egg Substitutes	10.8%			Fats and Oils	9.2%	
		Meat, Poultry, Their Products and Substitutes	10.7%			Cereals and Other Grain Products	8.5%	
		Marine and Fresh Water Animals	10.4%			Combination Dishes	8.0%	
		Bakery Products	9.7%			Meal Replacements	7.3%	
Calorie Specific Systems^g^	-			496	3.2%	Beverages	17.8%	-
*Systems that only display calorie or energy content.*						Fruit and Fruit juices	11.6%	
						Desserts	7.4%	
						Meal Replacements	7.3%	
						Salads	5.7%	

^a^Food categories defined as per Schedule M of the Food and Drug Regulations [13]

^b^Proportion of foods carrying each type of front-of-pack claim in FLIP 2010 (*n* = 10,487), as reported in Schermel et al., Appl Physiol Nutr Metab 2013;38(6):666-672 [24]

^c^Number of foods carrying each front-of-pack claim in FLIP 2010 were not included in this paper

^d^Number and proportion of foods carrying each type of front-of-pack claim in FLIP 2013 (*n* = 15,286)

^e^Proportion of foods carrying a front-of-pack claim in the top Schedule M food categories

^f^Statistical difference in the overall proportion of foods carrying front-of-pack claims between 2010 and 2013. A *p* value of <0.05 was considered statistically significant

^g^Calorie Specific Systems were introduced after 2010

b. **Other claims**

Regarding other claims, such as those related to intolerances and dietary practices, gluten-free claims were the only ones studied in the present research. Overall, gluten-free claims were displayed on 7.3% of pre-packaged food product labels. Detailed results will be published in a separate paper.

iii) **Trends in use of nutrition claims between 2010 and 2013**

- **Nutrition claims**

The use of claims in FLIP 2013 (49%) was comparable to the level seen in 2010 (48.1%) (χ^2^ = 2.4, df = 1, *p* = 0.115). When we excluded products that carried gluten-free claims to identify products with only nutrition claims, we found that 46.1% of FLIP 2013 foods carried any type of nutrient content claim, disease risk reduction claim and/or front-of-pack claim (χ^2^ = 9.5, df = 1, *p* = 0.002).

- ▪ *Nutrient content claims*

**Fig. 3:**
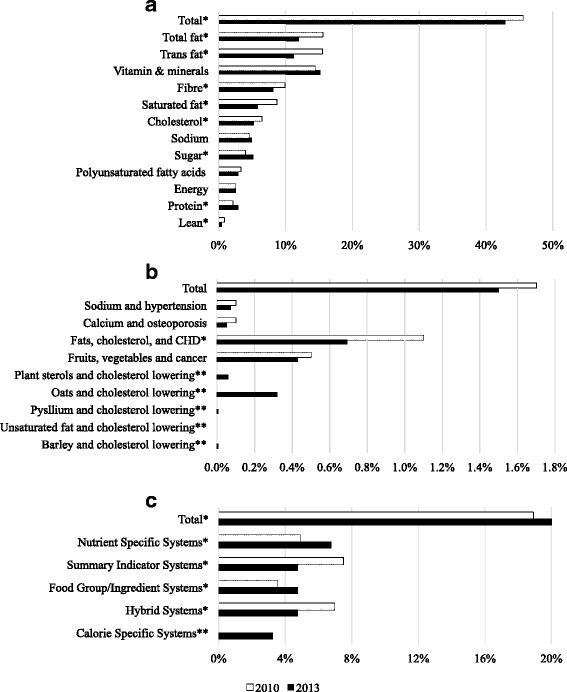
Trends in use of nutrition claims on Canadian packaged food products between 2010 and 2013. **a** Proportion of foods in FLIP 2010^1^ and FLIP 2013 with nutrient content claims, defined as “the amount of a nutrient or food constituent on a food” [13]. **b** Proportion of foods in FLIP 2010^1^ and FLIP 2013 with disease risk reduction claims, defined as “statements about the healthful effects of a certain food/food constituent consumed within a healthy diet on a person's health” [13]. **c** Proportion of foods with Front-of-pack (FOP) claims on the food package. FOP is defined as “systems that use nutrient criteria and symbols to indicate that a product has certain nutritional characteristics. Symbols are often placed on the principal display panel of the product, but may also be found on the side, top, or back panels or on self tags”) [24, 31] in FLIP 2010^1^ and FLIP 2013. FLIP data do not include retail administered on shelf FOP systems. 1. FLIP 2010 (Schermel et al., 2013) [24]. *Significant difference between 2010 and 2013 (*p* < 0.001).** Claims introduced in the market after 2010

Nutrient content claims were less used in 2013 (42.9%) when compared to 45.5% in 2010 (χ^2^ = 19.7, df = 1, *p* < 0.001) [24]. When claims referencing specific nutrients were analyzed (Fig. 3a), a significant decrease in the use of total fat (χ^2^ = 69.1, df = 1, *p* < 0.001), *trans* fat (χ^2^ = 101.2, df = 1, *p* < 0.001), fibre (χ^2^ = 23.1, df = 1, *p* < 0.001), saturated fat (χ^2^ = 75.9, df = 1, *p* < 0.001), cholesterol (χ^2^ = 16.3, df = 1, *p* < 0.001), and lean claims (χ^2^ = 14.1, df = 1, *p* < 0.001) were seen, while there was a significant increase in sugar (χ^2^ = 18.4, df = 1, *p* < 0.001), and protein claims (χ^2^ = 17.1, df = 1, *p* < 0.001). No significant changes in vitamin and mineral claims (χ^2^ = 2.7, df = 1, *p* = 0.095), sodium claims (χ^2^ = 2.4, df = 1, *p* = 0.121), omega-3 and omega-6 polyunsaturated fatty acids claims (χ^2^ = 3.15, df = 1, *p* = 0.076), and claims associated to energy (χ^2^ = 0.04, df = 1, *p* = 0.829) were seen.

- ▪ *Health claims*

The use of disease risk reduction claims did not change, with 1.5% carrying these claims in 2013 compared to 1.7% in 2010 (χ^2^ = 1.4, df = 1, *p* = 0.225) [24]. Claims related to saturated and *trans* fat and coronary heart disease continued to be the most prevalent disease risk reduction claim, although they were used significantly less often in 2013 when compared to 2010 (χ^2^ = 23, df = 1, *p* < 0.001) (Fig. 3b). Claims related to fruits, vegetables and cancer were used at similar levels compared to 2010 (χ^2^ = 1.15, df = 1, *p* = 0.283), as were disease risk reduction claims for sodium and hypertension (χ^2^ = 1.6, df = 1, *p* = 0.199) and calcium and osteoporosis (χ^2^ = 2.3, df = 1, *p* = 0.128). Claims related to oat products and blood cholesterol lowering (which were approved by Health Canada after 2010) were among the top three most frequently used disease risk reduction claims in 2013.

- ▪ *General health claims*

We specifically studied front-of-pack claims, which are a subtype of general health claims. Overall, the use of these claims was higher (20%) compared to 18.9% in FLIP 2010 (χ^2^ = 5.4, df = 1 *p* = 0.020) [24]. The trends among each subtype of front-of-pack were found as follows: the use of nutrient specific systems (χ^2^ = 39.9, df = 1, *p* < 0.001) and food group information systems were significantly increased (χ^2^ = 23, df = 1, *p* < 0.001), while summary indicator systems (χ^2^ = 84.3, df = 1, *p* < 0.001) and hybrid systems significantly decreased (χ^2^ = 55.8, df = 1, *p* < 0.001) (Fig. 3c). Calorie specific systems were introduced after 2010, and these types of front-of-pack claims accounted for 3.2% of all front-of-pack claims on food labels in 2013, which were especially prominent on beverages (see Table 4).

## Discussion

Overall, nutrition and other claims were frequently used on food labels in the Canadian food supply. Nutrition claims were used almost as often in 2013 as in 2010 [24]. Although, we expected the overall prevalence of nutrition claims (nutrient content claims, health claims, and general health claims within in the Canadian context), would be significantly higher in 2013 compared to 2010 due to changes in regulations and guidance documents mentioned earlier, this was not the case and in fact, it was lower. When specific types of nutrition claims were analyzed, the use of nutrient content claims significantly decreased, which could have driven the overall proportion of nutrition claims to decrease, as nutrient content claims were the most prevalent type of nutrition claims. The frequency of use of disease risk reduction claims not only remained low compared to nutrient content claims and front-of-pack claims, but also was unchanged and slightly lower compared to FLIP 2010 [24], despite the number of approved disease risk reduction claims being doubled in 2013 compared to 2010. Front-of-pack claims (a subtype of a general health claim) were used more often in 2013 and we found that other claims (specifically gluten-free claims) were used on foods labels in 2013.

In Canada, nutrient content claims can be used voluntarily by food manufacturers if products met the criteria established for each individual claim [13, 24]. The present study showed regulated nutrient content claims continued to be the type of nutrition claim most often used on food products (42.9%). Similar results were reported in the preceding study in Canada (45%) [24]. Trends among some individual types of nutrient content claims were identified. For instance, fat claims (total fat, *trans* fat and saturated fat) were less likely to be used in 2013 compared to 2010, consistent with a recent study that showed less emphasis is being made on fat in health messaging [36]. In that study, the authors noted that fat claims may be misleading consumers as they are not associated with lower calorie content in most foods, as most consumers expect [36]. Higher energy intake, rather than high fat per se, is probably one of the causes for obesity escalation [37]. Sodium claims were not significantly higher in 2013 compared to 2010 [24], despite huge efforts directed towards sodium reduction in Canada during this time [19]. For example, other research has shown that little sodium reduction progress overall has been achieved in the food supply during this period, although significant improvement has been achieved in some food categories [38]. One reason for the lack of low/reduced sodium claims could be that food manufacturers are using a step wise approach to reduce sodium in foods, as suggested by Health Canada’s sodium guidance document [19]; therefore, reductions are maybe not sufficient to reach the threshold of at least 25%, for a food to be allowed to carry a lower sodium nutrient content claim [13].

Only two types of nutrient content claims showed a significant increase between 2010 and 2013: sugar and protein claims. Although sugar claims were increasing in frequency, they are still only used approximately one third as often as nutrient content claims for fat or *trans* fat. Interest in sugar has risen in recent years and it is expected to continue to grow due the World Health Organization sugar guidelines which recommend keeping sugars, and particularly free sugars (defined as “all monosaccharides and disaccharides added to foods by the manufacturer, cook or consumer, plus sugars naturally present in honey, syrups and fruit juices”), to less than 10% of total energy intake [39]. Free sugars are associated with increased risk of dental caries, obesity, and type 2 diabetes. [39–41]. In 2016, the Government of Canada issued nutrition labelling regulatory changes, which included providing consumers with more information regarding sugar on food labels (e.g., a new daily value for total sugar and grouping sugars in the Ingredient List) [14]. However, as opposed to the United States [42], the change in labelling did not include adding free or added sugars on the Nutrition Facts table. Our research group has shown that free sugars account for approximately 20% of the calories in prepackaged foods and beverages in the Canadian food supply [25]. Thus, one could expect to find more products with sugar claims in the food supply in the upcoming years. With regards to protein claims, our study is consistent with a food trends report published in 2014, that showed 3% worldwide and 6% in the Unites States launches of food and beverage new products displaying either a “high-protein” or “source of protein” claim [43]. Also, the growth in the development of alternative and novel sources of protein [44], may provide new ingredients for new products with this nutrient.

Interestingly, disease risk reduction claims (a subtype of health claim) decreased slightly in frequency despite 5 new disease risk reduction claims being approved by Health Canada after 2010 [45–49]. However, this research is in line with results from studies in other countries that showed disease risk reduction claims were only present on 1-3% of food labels [3, 5, 50]. Nevertheless, another 6 disease risk reduction claims were approved between 2014 and 2016 (after data collection for this study) [51–56], which may result in an increase in their use by food manufacturers, although research has shown that disease risk reduction claims are not often used on food labels [5].

General health claims have a substantial presence in the Canadian food supply despite the fact they are not specifically regulated. As described in Fig. 1, front-of-pack claims are currently not regulated by Government, thus this may be one reason several systems were identified, which is far from the ideal single system recommended by nutrition experts [6]. Twenty percent of the products in FLIP 2013 carried front-of-pack claims, which is consistent with comparable research elsewhere [3]. Five different front-of-pack systems were identified on those food labels compared to 4 in 2010 [24], since an additional front-of-pack system related to calories was introduced after 2010, and used on 3.2% of food labels. The use of nutrient specific systems and food group/ingredient systems increased, while summary indicator systems and hybrid systems decreased. Nutrient specific systems were related primarily to single nutrients and very few products used a Guideline Daily Amounts (GDAs) or star rating system, and food group/ingredient systems were related mainly to whole grain. The decrease in use of summary indicators systems was largely due to the discontinuation of the Heart and Stroke Foundation’s Health Check™ program in 2013, which occurred during this collection [22]. Future collections of FLIP will likely reflect the complete termination of the Health Check program.

Besides the use of nutrition claims (nutrient content claims, health claims and other general health claims), this study also identified the frequency in use of other claims for the first time in our database, specifically gluten-free claims. This type of claim was present on 7.3% of the Canadian food supply, making it the fifth most popular claim, a proportion almost comparable to fibre claims (the fourth most common nutrient content claim), supporting reports that indicate “gluten-free claims” is a growing trend in food marketing in Canada [32, 57], perhaps largely driven because a number of non-celiac or non-gluten sensitive consumers are selecting gluten-free products because of their perceived “nutritional value” [32]. If this continues, one can expect products carrying gluten-free claims will be more prevalent in the upcoming years, although little research has been done to determine whether the nutritional value of these products is superior.

There are several strengths of this study. A major one is that this study provides a comprehensive assessment of the prevalence and trends in the use of nutrition marketing on foods in a structured way, using comparable methods as those established in the initial study conducted in 2010, which allowed us to objectively assess trends. This research also captured a large proportion of the products sold in the food supply in Canada (about 75% of the retail market); analyses did not restrict the selection to only certain food categories and certain types of claims. The use of electronic devices to photograph all foods in store allowed us to efficiently collect and process data. Although the categorization of some claims such as front-of-pack was not based on specific regulations, a standardized categorization framework was developed to minimize potential subjectivity, as it provided not only a path to decide whether claims fall into one or another category, but also provided graphic examples to guide those classifications.

Some limitations of this study were that it was cross-sectional in design. A second limitation is the approach used to classify nutrition claims. For example, this study classified both nutrient content claims and health claims as nutrition claims; however, other studies have used an international standardized nutrition labelling taxonomy to overcome the differences between regulations among countries [58–61], which can facilitate multi-country comparisons in the use of nutrition-related claims. FLIP 2013 did not capture products sold in value chain retailers, convenience stores and neighborhood stores, therefore some products available for purchase were probably missed, which might have included specialty products. Analyses were not weighted for sales data due cost restrictions, and the large sample size difference between 2010 and 2013 of products analyzed (*n* = 10,487 and *n* = 15,286 respectively), may have magnified variances in the claims assessed. However, to deal with this difference, analyses were carried out using weighted data (number of claims weighted to the number of products collected in each data set) rather than number of products collected.

In the end, different types of claims have been studied worldwide [3, 24, 58–63], although their impact on consumers’ health may be small [9]. Perhaps this is because claims are being used more as a marketing strategy by manufacturers [9]. However, monitoring and surveillance of the use of claims on food labels is important because it can help protect consumers from misleading information, evaluate regulatory compliance, provide information for public health research, or identify areas requiring improvement. Periodic evaluation of claims can also identify commonalities and differences among regions, which can be used for policy development and evaluation worldwide, support fair trade, among other uses.

## Conclusions

Nutrition and other claims were used on nearly half of Canadian prepackaged foods in 2013, like 2010. However, despite the release of new many claims guidelines and regulations since 2010, little impact has been seen on the prevalence of such claims in the food supply. Moreover, claims related to nutrients of public health priority, such as sugars and sodium, were not commonly used on food labels. Global efforts to monitor trends in the use of nutrition claims, and other food policies are underway [6, 60, 64]. Such efforts are essential to determine if the use of claims on food labels are reflecting the objectives of nutrition labelling to support healthy food choices [65]. Future studies should continue monitoring trends in the use of claims on food labels for regulatory surveillance and public health research, especially when updates to regulations are made and new science emerges.

## Abbreviations

CNF: Canadian nutrition file
CSS: Calorie specific systems
CVDs: Cardiovascular diseases
DRRC: Disease risk reduction claims
FDR: Food and Drug Regulations
FGIS: Food group/ingredient specific systems
FLIP: Food label information program
FOP: Front-of-pack claims
GDAs: Guideline daily amounts
HS: Hybrid systems
NCC: Nutrient content claims
NCDs: Non-communicable diseases
NSS: Nutrient specific systems
OCR: Optical character recognition
SIS: Summary indicator systems
St/Ft: Structure-Function claims
